# Supplementary material for: Pharmacologic inhibition of PCBP2 biomolecular condensates relieves Alzheimer’s disease
Source: Nat Commun. 2025 Nov 26;16:10514. doi: 10.1038/s41467-025-65547-9 (PMC12658114; doi:10.1038/s41467-025-65547-9)

Supplementary Information for
Pharmacologic inhibition of PCBP2 biomolecular condensates relieves Alzheimer's disease

Lu Wang[1,2,3,4,5#], Xiao-Yong Xie[1,2,3,4,5#], Qiu-Ling Pan[1,2,3,4,5], Jiawei Zhang[6], Gui-Feng Zhou[1,2,3,4,5], Qi-Lei Zhang[7], Xiao-Xin Yan[7], Yu Xiang[1,2,3,4,5], Chen-Lu Li[1,2,3,4,5], Yi He[2], Xiao-Jiao Xiang[8], Xiao-Juan Deng[1,2,3,4,5], Yan-Jiang Wang[9], Ji-Ying Zhou[1,2,3,4,5], Shenyou Nie[6], Guo-Jun Chen[1,2,3,4,5*]

[#] These authors contributed equally to this work.

* Corresponding author: 203165@cqmu.edu.cn (G-J Chen)

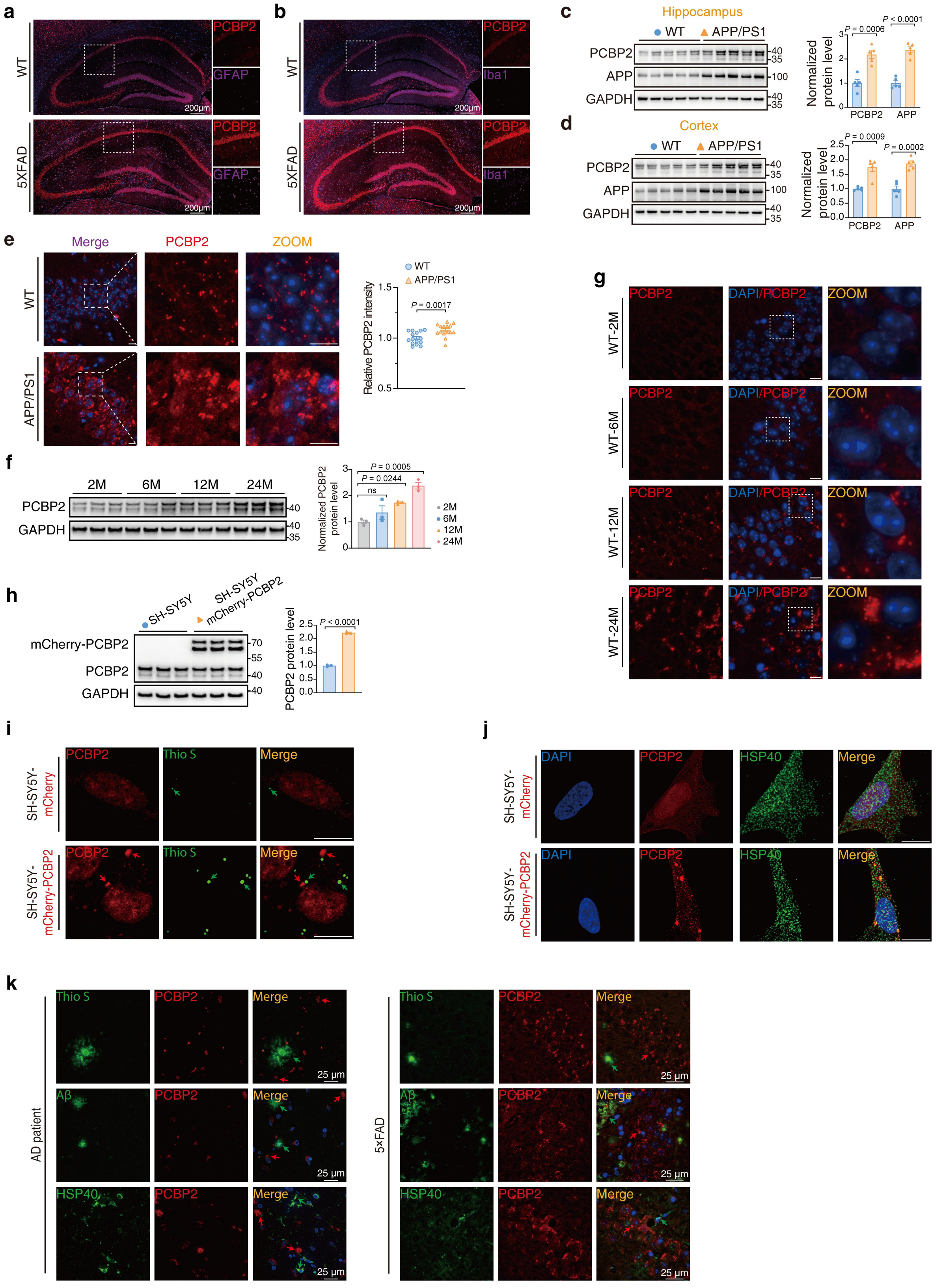

Supplementary Fig. 1 | Characterization of PCBP2 condensates. a, Representative immunofluorescence image of PCBP2 (Red), GFAP (Magenta) and DAPI (Blue) in the

hippocampal region of 12-month-old WT and 5×FAD. Scale bar, 200 µm. **b**, Confocal images of PCBP2 (red) and Iba1 (magenta)-immunostained hippocampal sections from 12-month-old WT and 5×FAD mice. Nuclear counterstaining was performed using DAPI (blue). Scale bar, 200 µm. **c**,**d**, Western blots (left) and quantification (right) of PCBP2 and APP in the hippocampus (**c**) and cortex (**d**) of APP/PS1 and WT mice. **e**, Representative immunofluorescence images (left) and quantification (right) of PCBP2 condensates (red) in the hippocampus of 12-month-old WT and APP/PS1 mice. Scale bar, 10 µm. **f**, **g**, Western blot analysis of PCBP2 (**f**) and representative images showing PCBP2 condensates (**g**, red) in the hippocampus of WT mice aged 2, 6, 12 and 24 months (M). Scale bar, 10 µm. **h**, Western blots (left) and corresponding quantification (right) of PCBP2 protein in SH-SY5Y cells and SH-SY5Y-mCherry-PCBP2 cells. **i**, **j**, Representative immunofluorescence images showing that mCherry-PCBP2 condensates (red) do not substantially colocalize with the Thioflavin S (ThioS, **i**) and aggresome marker HSP40 (**j**) in SH-SY5Y–mCherry-PCBP2 cells. Arrows in green: amyloid plaques or aggresome, red: PCBP2 condensates. Scale bar, 10 µm. **k**, Representative images showing that PCBP2 condensates do not markedly colocalize with aggresomes (HSP40) or amyloid plaques (ThioS and 6E10) in hippocampal sections from patients with Alzheimer's disease and 5×FAD mice. Arrows in green: aggregates or amyloid plaques, red: PCBP2 condensates. Scale bar, 25 µm. Data: mean ± s.e.m (**c**-**f**, **h**) and analyzed by two-tailed Student's t-test (**c**-**e**, **h**) and one-way ANOVA followed by Bonferroni's post-hoc test (**f**). ns: nonsignificant. In vitro: $n = 3$ biological replicates (**h**, **i**, **j**). In vivo: $n = 3$ (**a**, **b**, **f**, **g**, **k**); $n = 5$ (**c**-**e**). Source data are provided as a Source Data file.

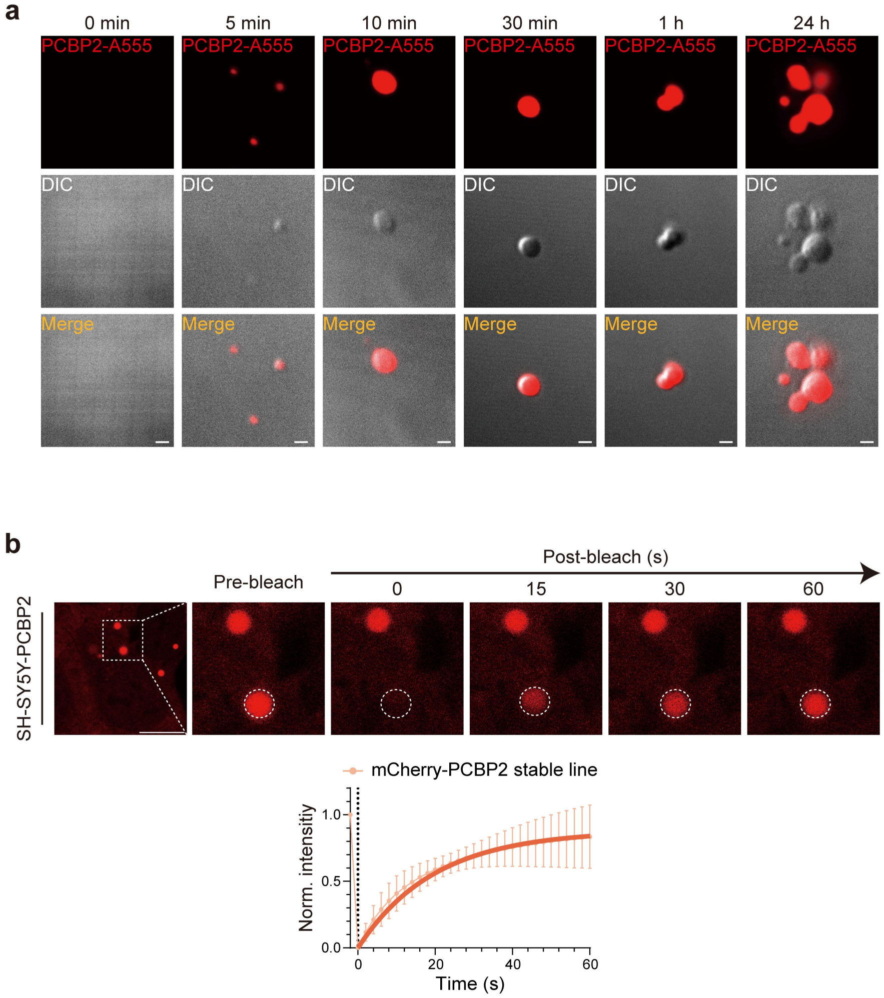

Supplementary Fig. 2 | Liquid-like dynamics of PCBP2 condensates. a, Confocal imaging of droplet formation in buffers containing RNA (20 ng/μL) and PCBP2 (2 μM), at 0, 5, 10, 30 min, 1 h and 24 h. Scale bar, 1 μm. **b**, Representative images (top) and quantification (bottom) show FRAP of mCherry-PCBP2 condensates in stably expressing mCherry-PCBP2 cells. The dashed box indicates the photobleached region (ROI). (*n* = 3 biological replicates). Scale bar, 10 μm. Data are presented as mean ± SD. Source data are provided as a Source Data file.

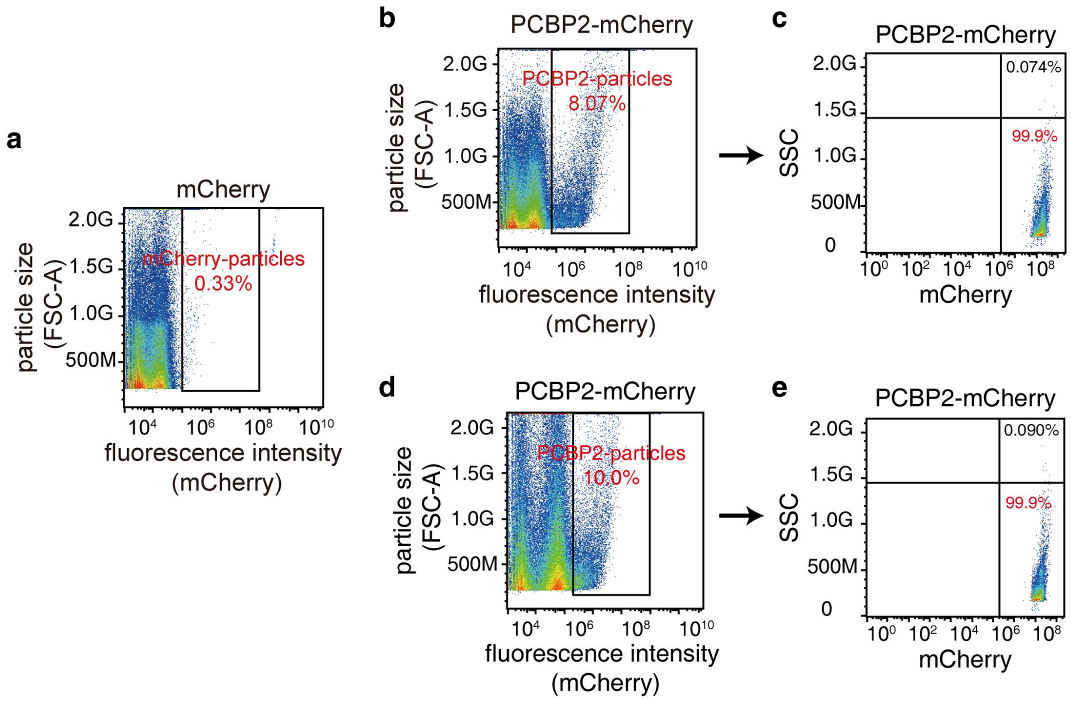

Supplementary Fig. 3 | FACS-based sorting of mCherry-PCBP2-positive particles. a-e, FAPS separated particles are based on their size and fluorescence. mCherry (**a**) serves as a control for non-PCBP2 particles. Sorting window for mCherry-PCBP2 particles in SH-SY5Y-mCherry-PCBP2 (**b**) and Post-sort fraction corresponding to the gate (**c**). Sorting window for mCherry-PCBP2 particles in SH-SY5Y-APP-mCherry-PCBP2 (**d**) and Post-sort fraction corresponding to the gate (**e**).

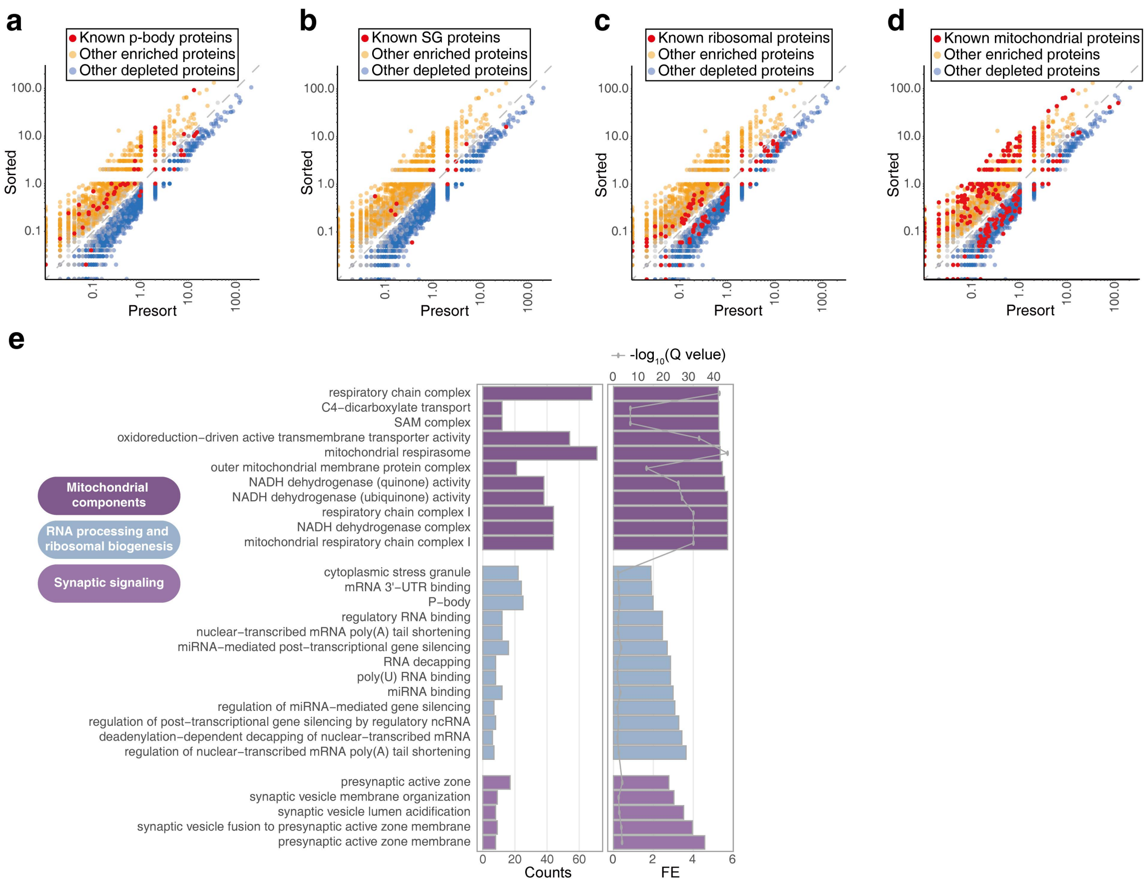

Supplementary Fig. 4 | Isolation and characterization of PCBP2 biomolecular condensates in SH-SY5Y-APP cells. a-d, Comparison of protein abundances between sorted and pre-sorted fractions utilizing quantitative values from normalized total spectra of LC-MS/MS. Significantly enriched and depleted proteins are indicated, alongside known p-body (**a**), SG proteins (**b**), ribosomal proteins (**c**), and mitochondrial proteins (**d**). **e**, Proteins identified by mass spectrometry were analyzed by gene ontology using the clusterProfiler with associated counts, fold enrichment (FE) values and Q-values.

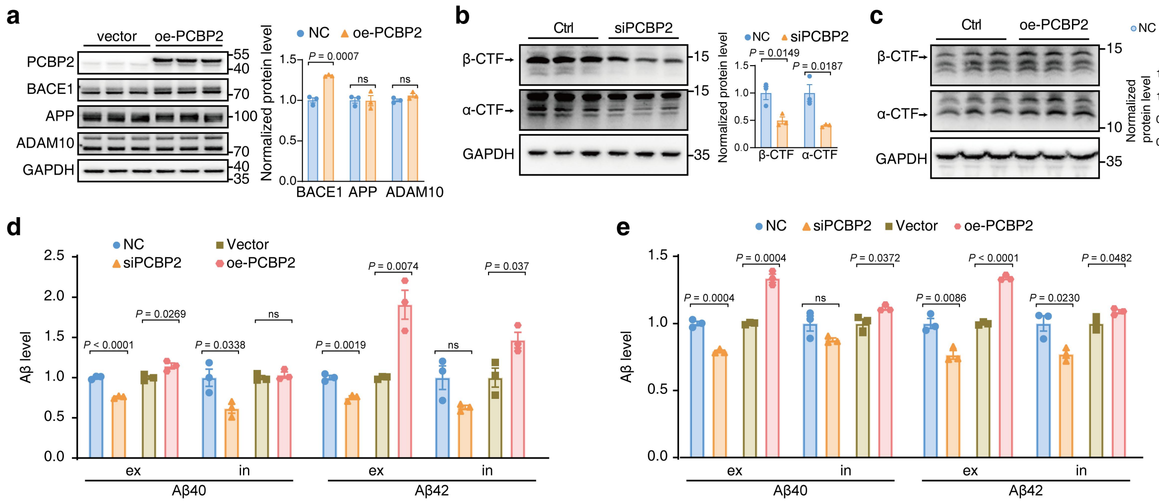

Supplementary Fig. 5 | PCBP2 condensates regulate BACE1 expression and Aβ load. a, Western blots (left) and quantification (right) on SH-SY5Y cells with overexpression of PCBP2. **b**,**c**, Representative Western blots (left) and quantification (right) of α/β-CTF in SH-SY5Y cells with knockdown PCBP2 (**b**) and overexpression of PCBP2 (**c**). **d**,**e**, Aβ40 and Aβ42 levels in culture medium (extracellular, ex) and cell lysates (intracellular, in) were measured using ELISA. SH-SY5Y-APP cells (**d**) and HEK293-APP cells (**e**) were treated with either PCBP2 knockdown (si-PCBP2) or PCBP2 overexpression (oe-PCBP2). Data: mean ± s.e.m (**a-f**) and analyzed by two-tailed Student's t-test (**a-f**). ns: nonsignificant. In vitro: $n = 3$ biological replicates (**a-f**). Source data are provided as a Source Data file.

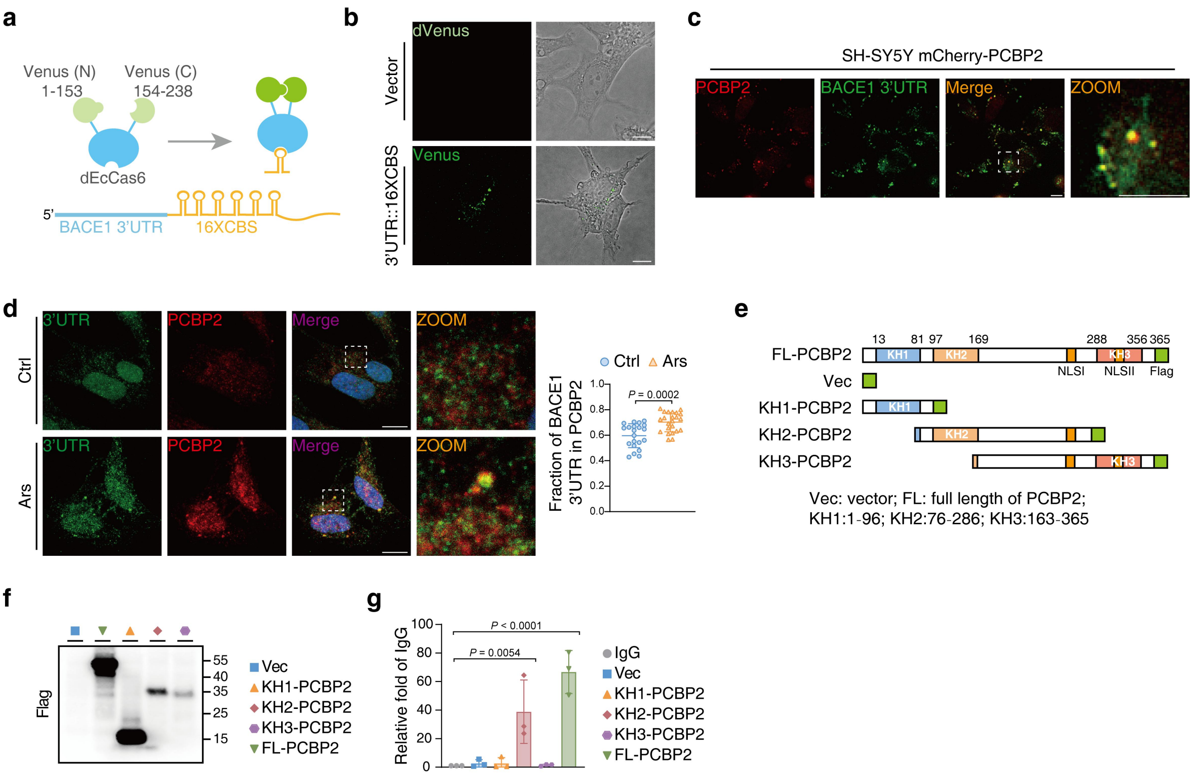

Supplementary Fig. 6 | PCBP2 binds to BACE1 3' UTR via the KH2 domain. a-c, Live-cell imaging of BACE1 3'UTR by fluorescence-activation platform. The system is partially based on dEcCas6, where Venus fluorescent protein is split into two inactive fragments linked to dEcCas6. Another component involves the tethering of the BACE1 3'UTR to a sequence comprising 16xCBS. Upon specific recognition of the CBS sequence by dEcCas6, a conformational change occurs, reactivating Venus fluorescent protein. This mechanism allows for the visualization of BACE1 mRNA in living cells. Schematic of the imaging system (**a**) utilized for BACE1 3'UTR visualization. Live-cell imaging of 3'UTR in SH-SY5Y cells (**b**) and SH-SY5Y mCherry-PCBP2 cells (**c**). Scale bars, 10 μm. **d**, Immunofluorescence images and their quantification demonstrate the colocalization of PCBP2 with an MS2-tagged 3'UTR reporter in SH-SY5Y cells cultured in the absence or presence of arsenite (Ars, 0.5 mM, 30 min). Scale bar, 10 μm. **e**, Schematic illustration of the full-length PCBP2 (FL-PCBP2) truncated into three segments, each retaining a single KH domain and tagged with a Flag epitope. **f**, Western blot analysis confirms the expression of these mutant proteins in eukaryotic cell. **g**, Relative BACE1 mRNA levels were measured by RNA immunoprecipitation (RIP) combined with RT-qPCR analysis. Data: mean ± SD (**d**, **g**) and analyzed by two-tailed Student's t-test (**d**) and one-way ANOVA with Dunnett's test (**g**). In vitro: $n = 3$ biological replicates (**b-d**, **f**, **g**). Source data are provided as a Source Data file.

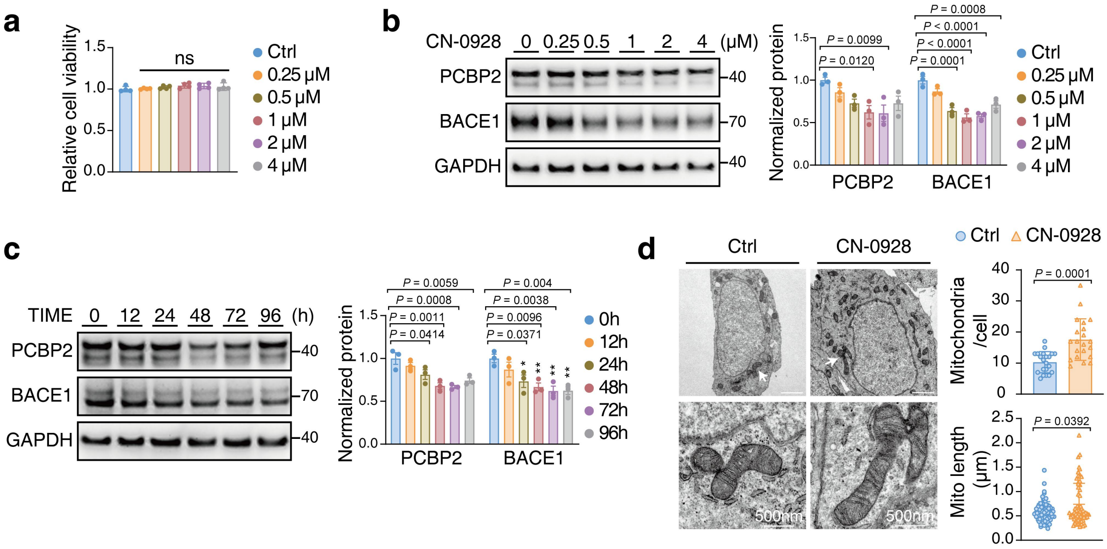

Supplementary Fig. 7 | CN-0928 reduces BACE1 and regains mitochondrial function in vitro. a, Relative cell viability of SH-SY5Y cells treated with indicated concentrations of CN-0928 (0 to 4 µM) for 48 h. **b**,**c**, Dose-response (**b**) and time-course (**c**) effect of CN-0928 on the protein levels of BACE1 and PCBP2 in SH-SY5Y cells. **d**, TEM images (left) of SH-SY5Y-PCBP2 cells treated with CN-0928 (1 µM, 48h). Lower panels display higher magnification of the areas highlighted by white arrows in upper panels. Scale bars: 2 µm or as indicated. Mitochondrial (mito) mean number and length, were quantified (right). Data: mean ± SD (**a**-**d**) and analyzed by one-way ANOVA with Dunnett's test (**a**-**c**); and Welch's t test (**d** [number]) and Kolmogorov-Smirnov test (**d** [length]). ns: nonsignificant. In vitro: $n = 3$ biological replicates (**a**-**d**). Source data are provided as a Source Data file.

a

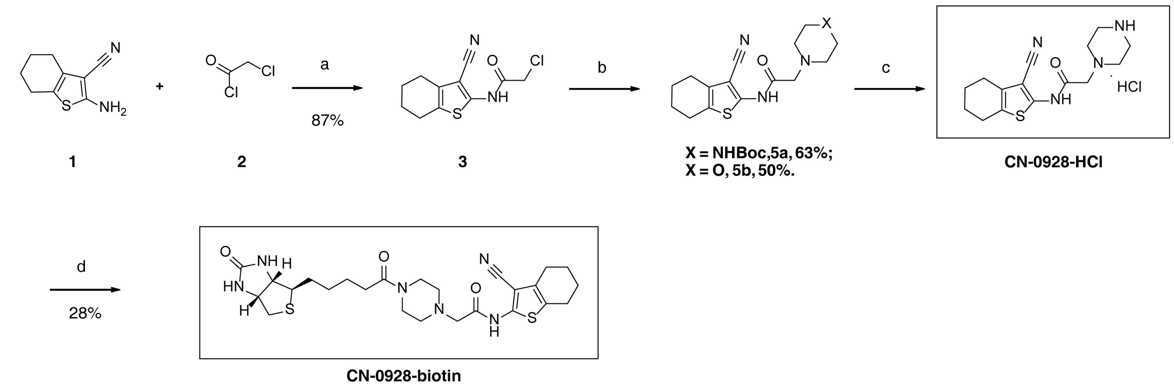
a
87%
b
c
d
28%
1
2
3
X = NHBoc,5a, 63%;
X = O, 5b, 50%.
CN-0928-HCl
CN-0928-biotin

b

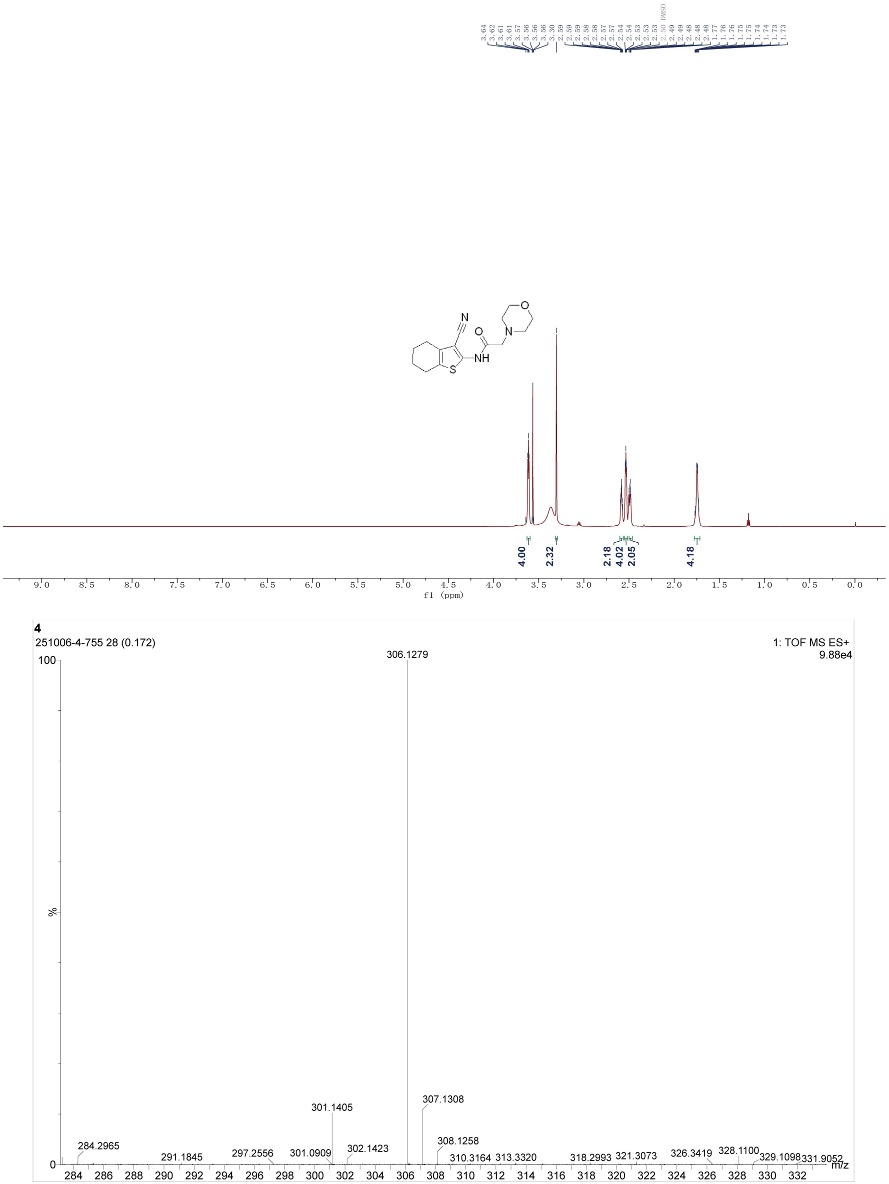
4.00
2.32
2.18
4.02
2.05
4.18
f1 (ppm)
4
251006-4-755 28 (0.172)
1: TOF MS ES+
9.88e4
306.1279
301.1405
307.1308
308.1258
284.2965
291.1845
297.2556
301.0909
302.1423
310.3164
313.3320
318.2993
321.3073
326.3419
328.1100
329.1098
331.9052
%
m/z

c

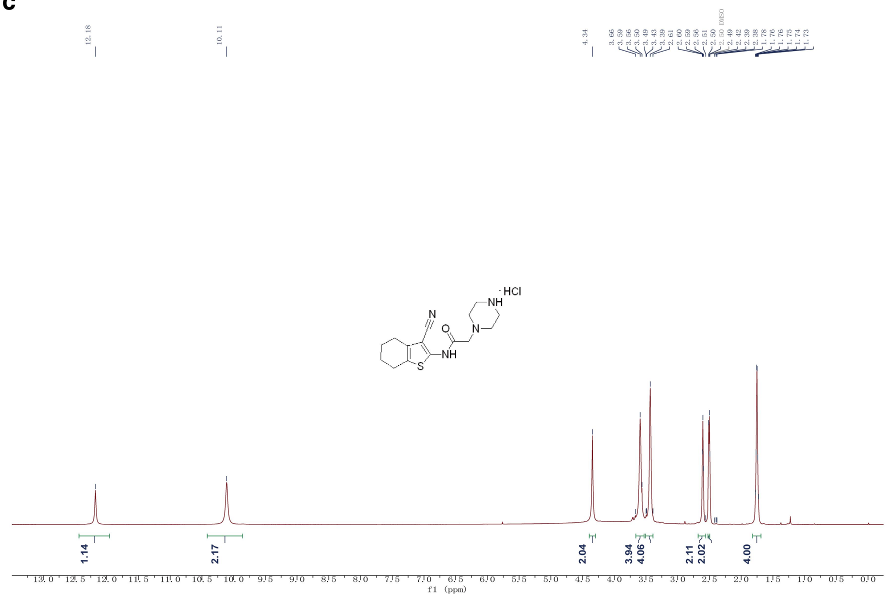
12.18
10.11
4.34
· HCl
NH
N
O
NH
S
1.14
2.17
2.04
3.94
4.06
2.11
2.02
4.00
f1 (ppm)

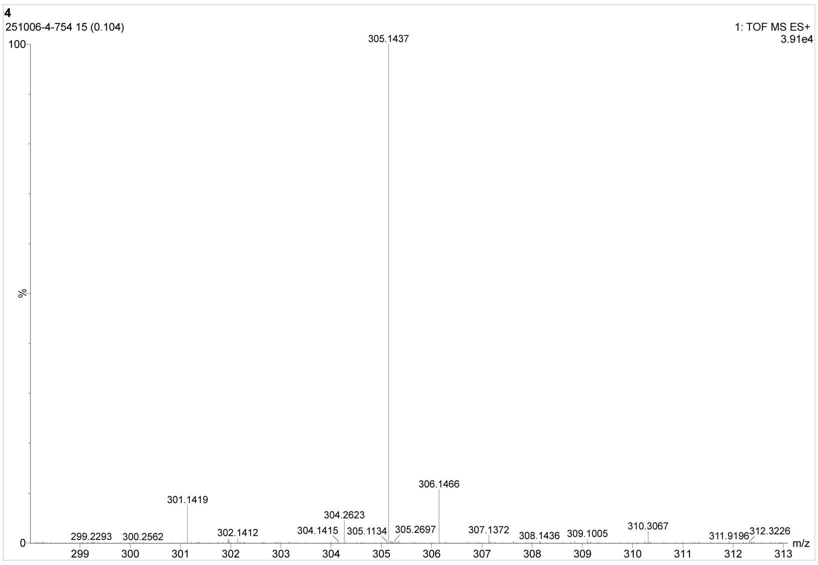
4
251006-4-754 15 (0.104)
1: TOF MS ES+
3.91e4
305.1437
306.1466
301.1419
304.2623
299.2293
300.2562
302.1412
304.1415
305.1134
305.2697
307.1372
308.1436
309.1005
310.3067
311.9196
312.3226
%
m/z

d

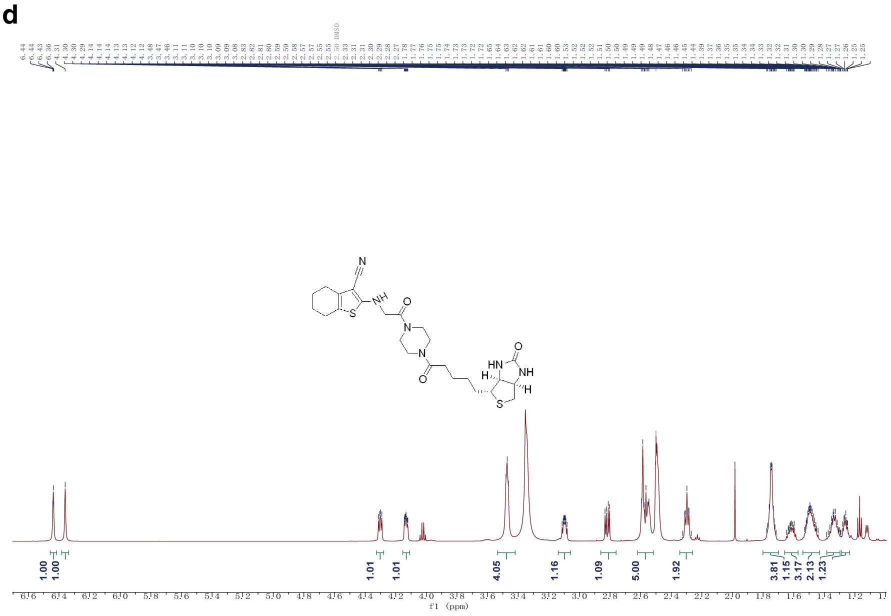

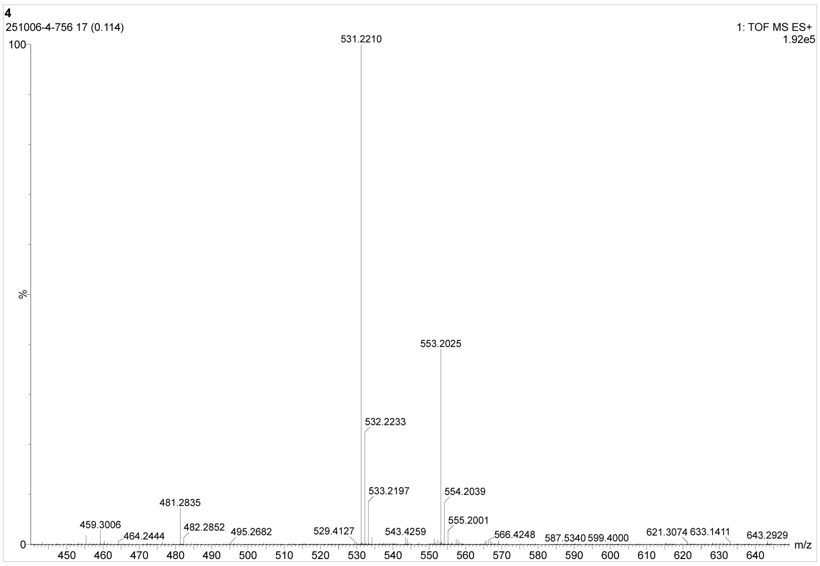

Supplementary Fig. 8 | Synthesis of CN-0928-biotin. a, Schematic diagram of the synthesis process for CN-0928-biotin. (a) 1,4-dioxane, rt, 30 min; (b) **4a-b**, TEA, 1,4-dioxane, 60 °C, overnight; **4a** = tert-butyl piperazine-1-carboxylate, **4b** = morpholine; (c) HCl (4 M in 1,4-dioxane), rt, 3 h; (d) **5**, HATU, DIPEA, DCM, rt, overnight; **5** = D-Biotin. **b**, ^{1}H NMR (600 MHz, DMSO-d_6) spectra of compound **5b**. ^{1}H NMR (600 MHz, DMSO-d$_6$) δ 3.65-3.58 (m, 4H), 3.30 (s, 2H), 2.58 (m, 2H), 2.56-2.52 (m, 4H), 2.52-2.45 (m, 2H), 1.75 (m, 4H). HRMS (ESI) calculated for $C_{15}H_{20}N_3O_2S^+$ ([M+H]$^+$): 306.1271, found 306.1279. **c**, ^{1}H NMR (600 MHz, DMSO-d_6) spectra of compound **CN-0928-HCl**. ^{1}H NMR (600 MHz, DMSO-d_6) δ 12.18 (s, 1H), 10.11 (s, 2H), 4.34 (s, 2H), 3.59 (s, 4H), 3.43 (s, 4H), 2.61 (d, J = 5.0 Hz, 2H), 2.53 – 2.48 (m, 2H), 1.76 (m, 4H). HRMS (ESI) calculated for $C_{15}H_{21}N_4OS^+$ ([M+H]$^+$): 305.1431, found 305.1437. **d**, ^{1}H NMR (600 MHz, DMSO-d_6) spectra of compound **CN-0928-biotin**. ^{1}H NMR (600 MHz, DMSO-d_6) δ 11.60-10.78 (s, 1H), 6.44 (s, 1H), 6.36 (s, 1H), 4.30 (m, 1H), 4.13 (m 1H), 3.47 (t, J = 5.4 Hz, 4H), 3.10 (m, 1H), 2.82 (dd, J = 12.6, 5.4 Hz, 1H), 2.57 (m, 5H), 2.34 –

2.26 (m, 2H), 1.74 (m, 5H), 1.62 (m, 1H), 1.54 – 1.43 (m, 4H), 1.34 (m, 2H), 1.26 (m, 1H). HRMS (ESI) calculated for $C_{25}H_{35}N_6O_3S_2^+$ ($[M+H]^+$): 531.2207, found 531.2210.

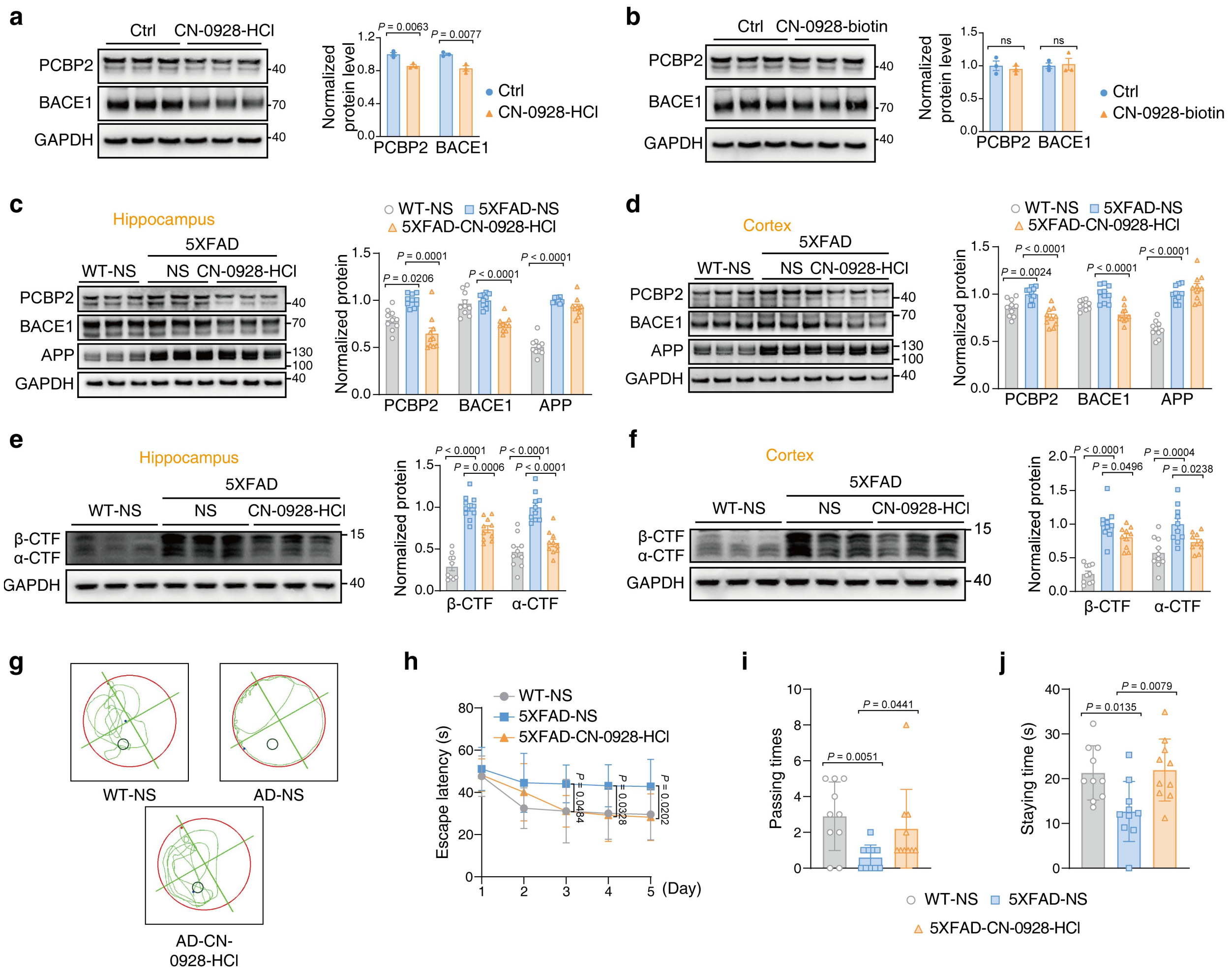

Supplementary Fig. 9 | CN-0928-HCl reduce amyloidogenesis and improves cognitive function in 5 × FAD mice. a,**b**, Western blots (left) and quantification (right) of PCBP2 and BACE1 in SH-SY5Y cells treated with CN-0928-HCl (**a**) or CN-028-biotin (**b**). **c**-**j**, Male 5×FAD mice were i.p. injected with 3.5 mg/kg CN-0928-HCl every other day for one month. In this study, WT mice injected with NS (WT-NS) and 5×FAD mice injected with NS (5×FAD-NS) represent the same mice (also described in major Figure 6). **c**,**d**, Representative Western blots (left) and quantification (right) of PCBP2, BACE1, and APP protein levels in the hippocampus (**c**) and cortex (**d**). **e**,**f**, Representative Western blots (left) and quantification (right) of α/β-CTFs levels in the hippocampus (**e**) and cortex (**f**). **g**, Representative trajectory maps for three groups of mice in the probe trial. **h**, Mice treated with CN-0928-HCl exhibited a shorter escape latency compared to those treated with NS. **i**, The frequency for the mice passing through the platform in the spatial probe test. **j**, The staying time in the target quadrant. Data: mean ± s.e.m (**a**-**f**) and mean ± SD (**h**-**j**). Significance was determined by the unpaired two-tailed Student's t test (**a**, **b**) and one-way ANOVA followed by Bonferroni's post-hoc test (**c**-**f**, **h**, **j**), and Welch's one-way ANOVA with Dunnett's T3 post hoc test; Brown-Forsythe variance test (**i**). ns: nonsignificant. In vitro: $n = 3$ biological replicates (**a**, **b**). In vivo: $n = 10$ (**c**-**f**, **h**-**j**). Source data are provided as a Source Data file.

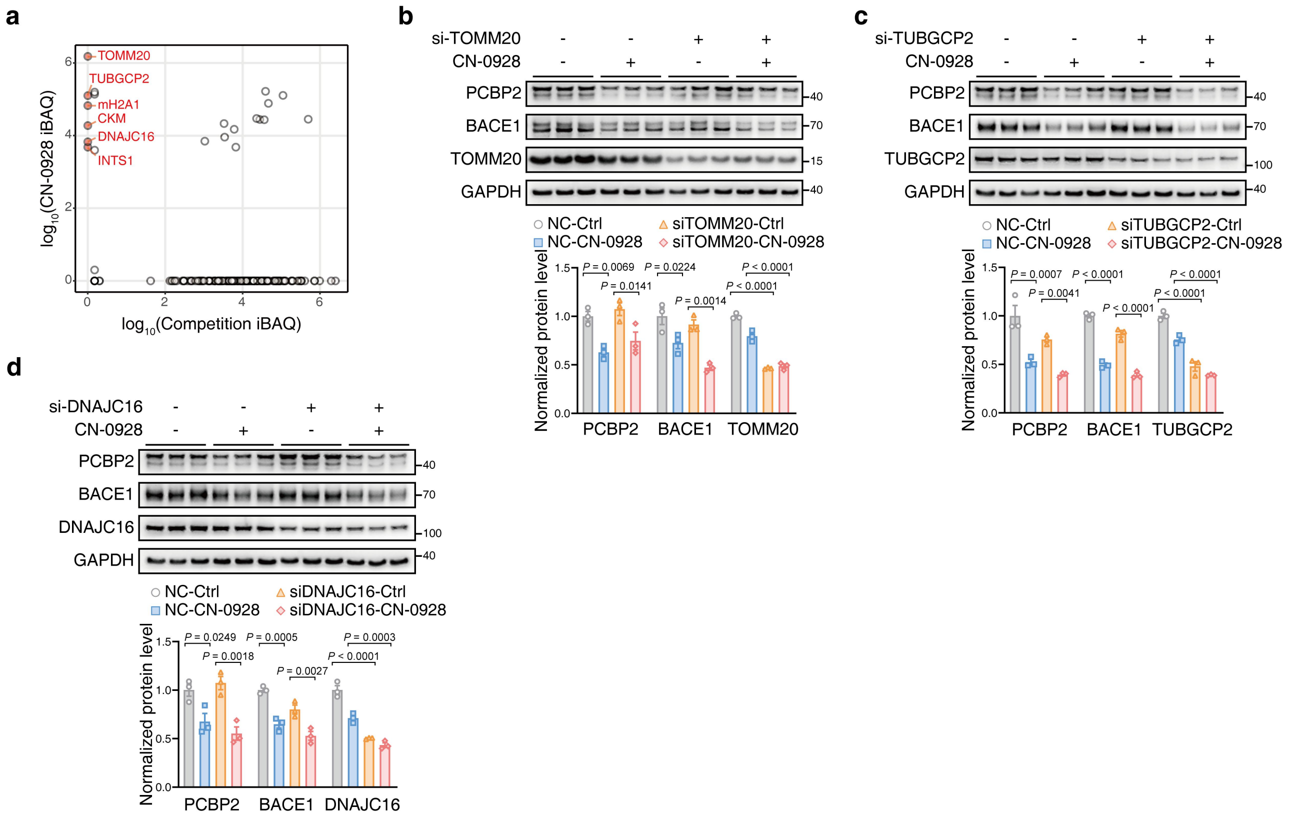

Supplementary Fig. 10 | Ligandability analysis and functional validation of additional CN-0928 target proteins. **a**, Scatter plot of ligandability displaying iBAQ values obtained with CN-0928-biotin alone plotted against those obtained after competition with free CN-0928. Proteins highlighted in orange were detected in both replicates of the CN-0928 group but not in the competition group or the blank control. **b-d**, Representative western blots (upper) and quantification (lower) of PCBP2 and BACE1 in SH-SY5Y cells transiently transfected with siRNAs against TOMM20 (**b**, siTOMM20), TUBGCP2 (**c**, siTUBGCP2) or DNAJC16 (**d**, siDNAJC16) and treated with 1 μM CN-0928 or DMSO (Ctrl). Data: mean ± s.e.m (**b-d**). Significance was determined by one-way ANOVA followed by Bonferroni's post-hoc test (**b-d**). In vitro: $n = 3$ biological replicates (**b-d**). Source data are provided as a Source Data file.

Supplementary Table 1.

Postmortem human brain samples used in this study.

Case #	Age (yrs)	Sex	Clinical diagnosis or cause of death	Tauopathy (Braak)	Amyloid pathology (Thal)
Cases used for pathological and immunoblot characterization					
P1	88	F	AD, CHD, DM, pneumonia	IV	5
P2	83	F	Demented, AMI	IV	3
P3	88	F	Demented, hypertension	V	4
P4	95	M	AD, Heart failure, DM	IV	1
P5	86	M	Demented, CHD	III	4
P6	87	F	AD, CHD, DM	II	0
P7	91	F	AD, CHD, hypertension, DM	VI	5
P8	86	F	AD, respiratory failure,CHD	IV	1
P9	92	F	Demented, respiratory failure, CHD, hypertension, DM	III	1
P10	83	M	Demented, respiratory failure	V	4
Cases used for comparative pathological characterization					
C1	86	M	Heart failure, hypertension, CHD	II	1
C2	92	F	Lung cancer, CHD, heart failure	VI	3
C3	64	M	Gastric cancer	II	1
C4	75	F	Respiratory failure	III	2
C5	65	M	Esophageal cancer	-	0
C6	83	M	Heart failure, CHD	III	2
C7	76	M	Liver cancer	I	0
C8	89	M	Heart failure, hypertension	II	3
C9	79	M	Heart failure, CHD, hypertension, lacunar infarction	III	4
C10	81	M	Gastrointestinal bleeding	II	1

AD, alzheimer disease; CHD, coronary heart disease; AMI, acute myocardial infarction; DM, diabetes mellitus.

Supplementary Table 2. Small molecule screening data

Category	Parameter	Description
Assay	Type of assay	cell-based
	Target	PCBP2
	Primary measurement	Relative PCBP2 intensity
	Key reagents	PCBP2 [EPR14858] (mAb, Abcam, catalog no. ab184962)
	Assay protocol	Live-cell imaging and immunofluorescence, and Western blotting sections in Methods.
	Additional comments	None
Library	Library size	3000
	Library composition	drug-like molecules
	Source	TOPSCIENCE
	Additional comments	None
Screen	Format	96-well plates
	Concentration(s) tested	1 μM compound, 0.1% DMSO
	Plate controls	Controls: negative = 0.1% DMSO
	Reagent/ compound dispensing system	Eppendorf manual pipettes (0.1-100 μL), manual dispensing into assay plates.<br>Compounds and controls prepared in 0.1% DMSO (final), using sterile, filtered tips.
	Detection instrument and software	Leica THUNDER Imager Live Cell with integrated THUNDER Analysis module for image acquisition and analysis.
	Assay validation/QC	Single-control assay; negative control = 0.1% DMSO. Z' not applicable. Plate acceptance required DMSO CV ≤ 10% and S/B (DMSO vs blank/no-cell) ≥ 5.
	Correction factors	Per-plate background subtraction using blank/no-cell wells.
	Normalization	%Activity = 100×(signal−blank)/(DMSO−blank)<br>%Inhibition = 100−%Activity
	Additional comments	None
Post-HTS analysis	Hit criteria	Required %Inhibition ≥ 50%.
	Hit rate	Initial hits: [28]/[3000] ([0.93]%). Western blot confirmed: [2]/[28] ([7.14]%).
	Additional assay(s)	Western blotting
	Confirmation of hit purity and structure	Hits were repurchased from [TOPSCIENCE] where available. Identity was confirmed by LC-MS.
	Additional comments	None

Supplementary Figure 1c

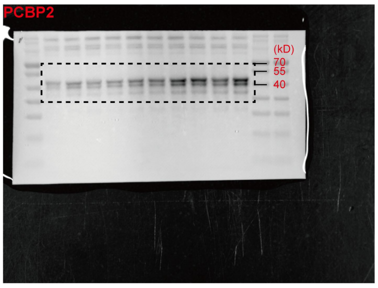

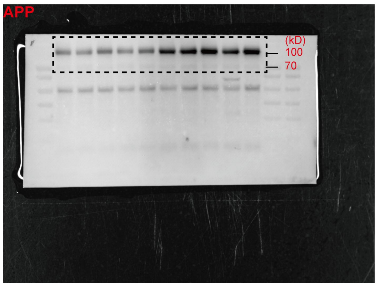

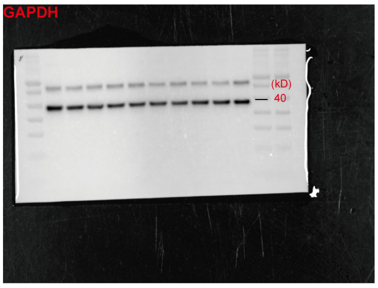

Supplementary Figure 1d

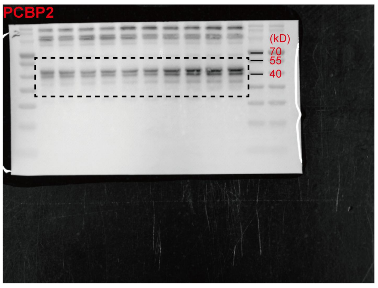

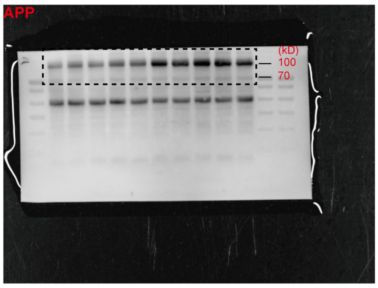

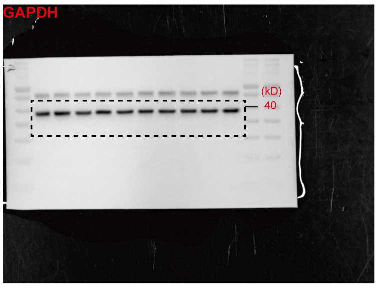

Supplementary Figure 1f

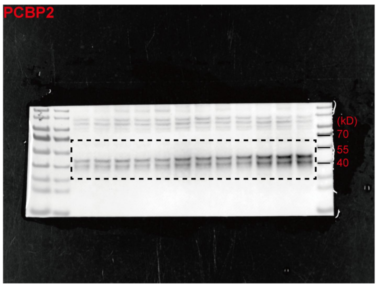

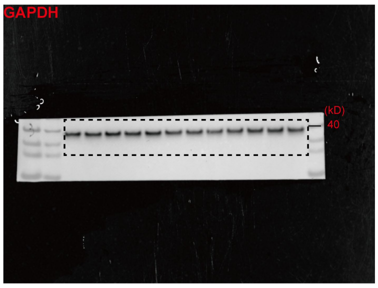

Supplementary Figure 1h

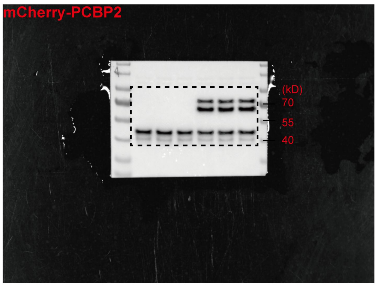

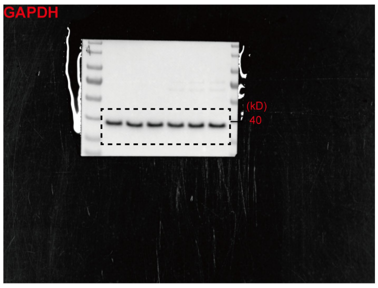

Supplementary Figure 5a

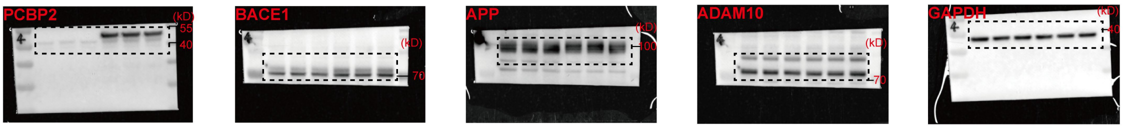

Supplementary Figure 5b

β-CTF

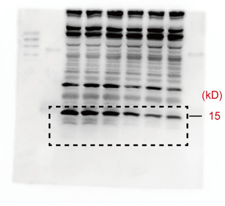

α-CTF

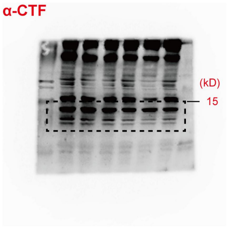

GAPDH

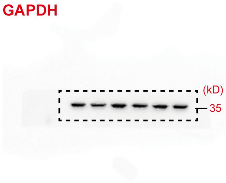

Supplementary Figure 5c

β-CTF

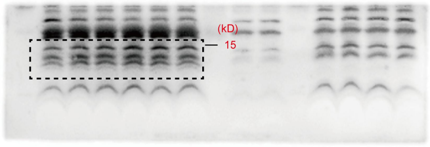

GAPDH

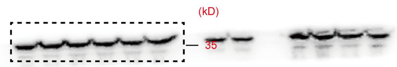

α-CTF

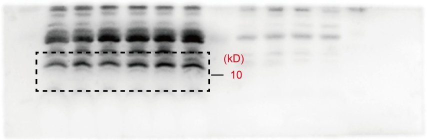

Supplementary Figure 6f

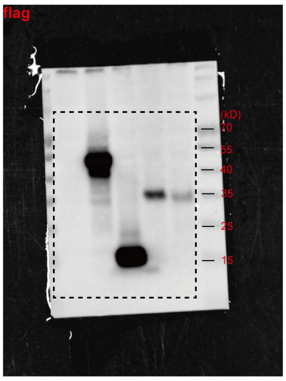

Supplementary Figure 7b

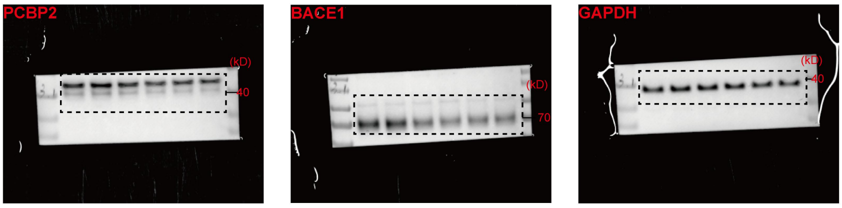

Supplementary Figure 7c

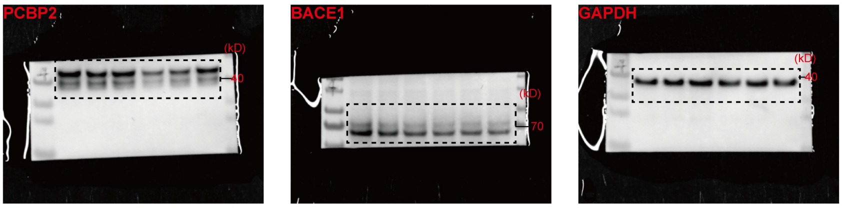

Supplementary Figure 9a

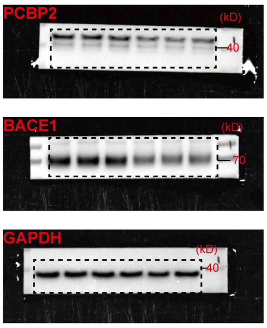

Supplementary Figure 9b

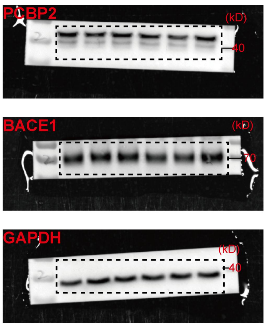

Supplementary Figure 9c

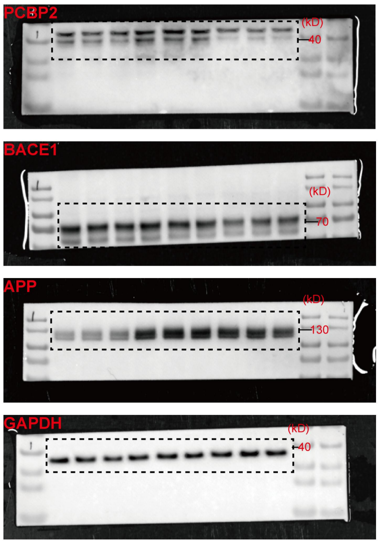

Supplementary Figure 9d

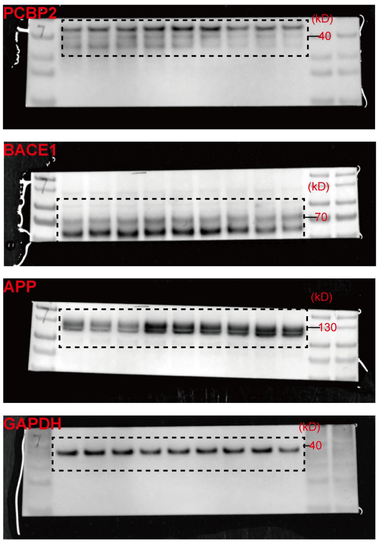

Supplementary Figure 9e

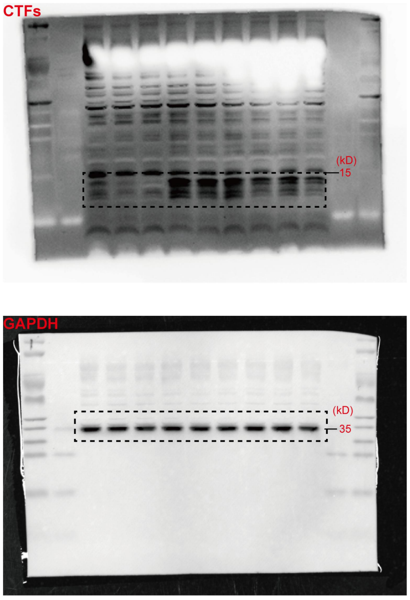

Supplementary Figure 9f

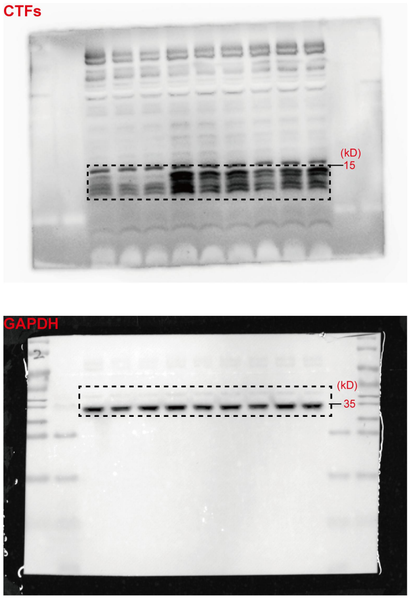

Supplementary Figure 10b

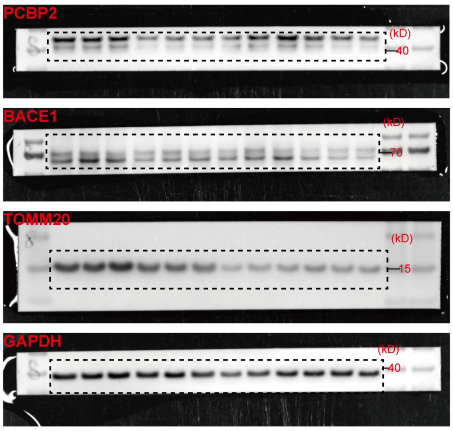

Supplementary Figure 10c

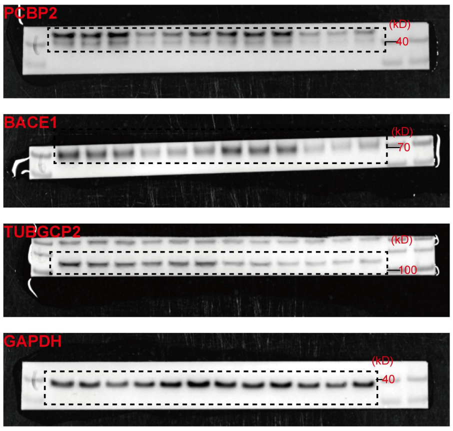

Supplementary Figure 10d

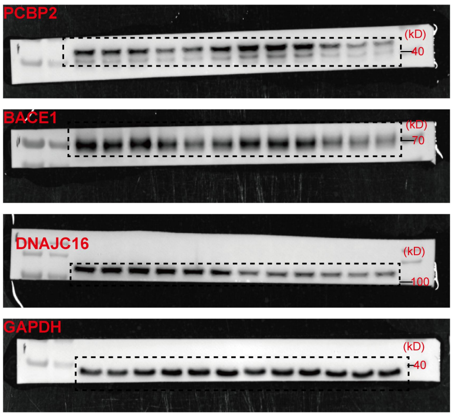

Supplement: Supplementary file 1 — Supplementary Information [file 41467_2025_65547_MOESM1_ESM.pdf]
