# Supplementary material for: Pharmacologic inhibition of PCBP2 biomolecular condensates relieves Alzheimer’s disease
Source: Nat Commun. 2025 Nov 26;16:10514. doi: 10.1038/s41467-025-65547-9 (PMC12658114; doi:10.1038/s41467-025-65547-9)
Supplement: Supplementary file 2 — Description of Additional Supplementary Files [file 41467_2025_65547_MOESM2_ESM.pdf]

Description of Additional Supplementary Files

Title: Supplementary Movie 1

Description: PCBP2 condensates underwent dynamic fusion in living cells.

Title: Supplementary Movie 2

Description: PCBP2 condensates were inhibited by 1,6-hexanediol.

Title: Supplementary Data 1

Description: Analysis of PCBP2 condensate components in SH-SY5Y-mCherry-PCBP2 cells.

Title: Supplementary Data 2

Description: clusterProfiler-based functional enrichment results derived from SH-SY5Y-mCherry-PCBP2 cells following PCBP2 condensate sorting.

Title: Supplementary Data 3

Description: clusterProfiler-based functional enrichment results derived from SH-SY5Y-APP-mCherry-PCBP2 cells following PCBP2 condensate sorting.

Title: Supplementary Data 4

Description: RNA pull-down coupled with LC-MS/MS using the BACE1 3’UTR as bait.

Title: Supplementary Data 5

Description: This dataset lists proteins captured by CN-0928-conjugated beads (CN-0928 group), enabling identification of proteins enriched as candidate CN-0928 targets.

Title: Supplementary Data 6

Description: All plasmids, siRNAs, and primers used in this study.
