# Supplementary material for: Pharmacologic inhibition of PCBP2 biomolecular condensates relieves Alzheimer’s disease
Source: Nat Commun. 2025 Nov 26;16:10514. doi: 10.1038/s41467-025-65547-9 (PMC12658114; doi:10.1038/s41467-025-65547-9)
Supplement: Supplementary file 11 — Reporting Summary [file 41467_2025_65547_MOESM11_ESM.pdf]

nature portfolio

Corresponding author(s): Guo-Jun Chen

Last updated by author(s): Oct 1, 2025

Reporting Summary

Nature Portfolio wishes to improve the reproducibility of the work that we publish. This form provides structure for consistency and transparency in reporting. For further information on Nature Portfolio policies, see our Editorial Policies and the Editorial Policy Checklist.

Statistics

For all statistical analyses, confirm that the following items are present in the figure legend, table legend, main text, or Methods section.

n/a	Confirmed	
☐	☒	The exact sample size (*n*) for each experimental group/condition, given as a discrete number and unit of measurement
☐	☒	A statement on whether measurements were taken from distinct samples or whether the same sample was measured repeatedly
☐	☒	The statistical test(s) used AND whether they are one- or two-sided<br>*Only common tests should be described solely by name; describe more complex techniques in the Methods section.*
☐	☒	A description of all covariates tested
☐	☒	A description of any assumptions or corrections, such as tests of normality and adjustment for multiple comparisons
☐	☒	A full description of the statistical parameters including central tendency (e.g. means) or other basic estimates (e.g. regression coefficient) AND variation (e.g. standard deviation) or associated estimates of uncertainty (e.g. confidence intervals)
☐	☒	For null hypothesis testing, the test statistic (e.g. *F*, *t*, *r*) with confidence intervals, effect sizes, degrees of freedom and *P* value noted<br>*Give P values as exact values whenever suitable.*
☒	☐	For Bayesian analysis, information on the choice of priors and Markov chain Monte Carlo settings
☒	☐	For hierarchical and complex designs, identification of the appropriate level for tests and full reporting of outcomes
☐	☒	Estimates of effect sizes (e.g. Cohen's *d*, Pearson's *r*), indicating how they were calculated

Our web collection on statistics for biologists contains articles on many of the points above.

Software and code

Policy information about availability of computer code

Data collection	All data comparisons were conducted using GraphPad Prism version 9.5.1. Fluorescence images were acquired using LAS X (Leica), with the LIGHTNING module employed for super-resolution imaging and the THUNDER module for defocus elimination. Flow cytometry was collected and analyzed with SQ Software (Invitrogen) and FlowJo version 10.8.1, respectively. Metabolic analysis data were acquired and processed using Wave software version 2.6.1.53 (Agilent). Molecular docking experiments were conducted using MOE2019, and Gromacs2018 was used as the molecular dynamics simulation software.
Data analysis	In addition to software above, mass spectrometry data were analysed in R Version 4.3.1. Western blot and fluorescence images were analyzed using ImageJ.

For manuscripts utilizing custom algorithms or software that are central to the research but not yet described in published literature, software must be made available to editors and reviewers. We strongly encourage code deposition in a community repository (e.g. GitHub). See the Nature Portfolio guidelines for submitting code & software for further information.

Data

Policy information about availability of data

All manuscripts must include a data availability statement. This statement should provide the following information, where applicable:

- Accession codes, unique identifiers, or web links for publicly available datasets
- A description of any restrictions on data availability
- For clinical datasets or third party data, please ensure that the statement adheres to our policy

Unless otherwise stated, all data supporting the results of this study can be found in the article, supplementary, and source data files. RNA sequencing data of PCBP2 knockdown in SH-SY5Y cells have been deposited in the Gene Expression Omnibus (GEO) under the accession numbers GSE306042 [https://www.ncbi.nlm.nih.gov/geo/query/acc.cgi?acc=GSE306042]. The mass spectrometry proteomics data have been deposited to the ProteomeXchange Consortium via the iProX partner repository with the dataset identifier PXD055459 [https://www.iprox.cn/page/project.html?id=IPX0009568000]. Raw spectral characterization data for all synthesized small molecules have been deposited in Figshare and are publicly available at [https://doi.org/10.6084/m9.figshare.29972815]. Source Data are provided with this paper.

Research involving human participants, their data, or biological material

Policy information about studies with human participants or human data. See also policy information about sex, gender (identity/presentation), and sexual orientation and race, ethnicity and racism.

Reporting on sex and gender	1. The sex of every human brain donor included in the study has been reported in Supplementary Table 1.<br>2. Regarding the study involving human brain tissue, sex and/or gender were not considered in the study design. This information is now clearly mentioned in Supplementary Table 1.
Reporting on race, ethnicity, or other socially relevant groupings	Not collected-unavailable from the brain bank.
Population characteristics	Detailed characteristics of human brain tissues used in this study, including age, sex, diagnosis are described in Supplementary Table 1.
Recruitment	Samples of pre-existing human autopsy brain with no personal identifiers were sourced from the Human Brain Bank at Xiangya School of Medicine, obtained through its voluntary donation program.
Ethics oversight	Postmortem human brains utilized in this study were sourced from the Human Brain Bank at Xiangya School of Medicine, obtained through its voluntary donation program. This project was approved by the Institutional Review Board of School of Basic Medical Science, Central South University (approval no. 2020KT37). The study was conducted in compliance with the requirements of related regulations and procedures (such as Ethical Review of Biomedical Research Involving Human Subject, ICH-GCP) as well as the ethical principles and followed the Standardized Operational Protocol established by the China Human Brain Banking.

Note that full information on the approval of the study protocol must also be provided in the manuscript.

Field-specific reporting

Please select the one below that is the best fit for your research. If you are not sure, read the appropriate sections before making your selection.

[x] Life sciences [ ] Behavioural & social sciences [ ] Ecological, evolutionary & environmental sciences

For a reference copy of the document with all sections, see nature.com/documents/nr-reporting-summary-flat.pdf

Life sciences study design

All studies must disclose on these points even when the disclosure is negative.

Sample size	The sample size was estimated based on our previous experience on the model. No sample size calculation was done either for in vivo or in vitro studies. For in vivo studies, n = 5-10 mice per group is sufficient to detect meaningful biological differences with good reproducibility. For in vitro studies, all the experiments were replicated at least for 3 individual, independent experiments.
Data exclusions	No data were excluded from the manuscript.
Replication	All experiments in this manuscript were repeated three times, and all experiments were reproducible. All data presented are biological replicates unless otherwise stated in the figure legends.
Randomization	All samples in this manuscript were randomly assigned to the experimental group.
Blinding	The experiments were not performed in a blinded manner as the investigator needed to know the treatment groups in order to complete the study. All data were acquired and analyzed by software with objective standard, thus blinding was not relevant to the study.

Reporting for specific materials, systems and methods

We require information from authors about some types of materials, experimental systems and methods used in many studies. Here, indicate whether each material, system or method listed is relevant to your study. If you are not sure if a list item applies to your research, read the appropriate section before selecting a response.

Materials & experimental systems

n/a	Involved in the study
☐	☒ Antibodies
☐	☒ Eukaryotic cell lines
☒	☐ Palaeontology and archaeology
☐	☒ Animals and other organisms
☒	☐ Clinical data
☒	☐ Dual use research of concern
☒	☐ Plants

Methods

n/a	Involved in the study
☒	☐ ChIP-seq
☐	☒ Flow cytometry
☒	☐ MRI-based neuroimaging

Antibodies

Antibodies used

Antibodies against the following proteins or epitopes were purchased from the indicated sources: ADAM10 (pAb, Abcam, catalog no. ab1997, 1:1000); APP, C-Terminal (pAb, Sigma, catalog no. A8717, 1:1000); β-Amyloid, 1-16 [6E10] (mAb, BioLegend, catalog no. 803014, 1:500); BACE1 (pAb, Abcam, catalog no. ab2077, 1:1000); BACE1 [EPR3956] (mAb, Abcam, catalog no. ab108394, 1:2000); BACE1 [EPR19523] (mAb, Abcam, catalog no. ab183612, 1:200); PCBP2 [EPR14858] (mAb, Abcam, catalog no. ab184962, 1:1000 for WB; 1:200 for IF); RENT1/hUPF1 [EPR4681] (mAb, Abcam, catalog no. ab109363, 1:1000); phospho-Upf1 (Ser1127) (pAb, Sigma, catalog no. 07-1016, 1:1000); TIA1 (mAb, Santa Cruz, catalog no. sc-166247, 1:50); DDX6 (mAb, Santa Cruz, catalog no. sc-376433, 1:50); DCP1a (mAb, Santa Cruz, catalog no. sc-100706, 1:50); TOMM20 [EPR15581-39] (mAb, Abcam, catalog no. ab283317, 1:1000 for WB; 1:200 for IF); NeuN [1B7] (mAb, Abcam, catalog no. ab104224, 1:200); Iba1 [EPR16589] (mAb, Abcam, catalog no. ab283346, 1:200); GFAP [EPR1034Y] (mAb, Abcam, catalog no. ab279291, 1:200); INTS1(pAb, Proteintech, catalog no. 31428-1-AP, 1:1000); Histone Macro-H2A.1 (mAb, Invitrogen, catalog no. MA5-24696, 1:1000); TUBGCP2 (pAb, Proteintech, catalog no. 25856-1-AP, 1:1000); HSP40 (mAb, Santa Cruz, catalog no. sc-398766, 1:50); DNAJC16 (pAb, FineTest, catalog no. FNab02461, 1:1000); Flag [DYKDDDDK tag] (mAb, Proteintech, catalog no. 66008-4-Ig, 1:1000); GFP (mAb, Proteintech, catalog no. 66002-1-Ig, 1:1000); mCherry (mAb, Proteintech, catalog no. 68088-1-Ig, 1:2000); GAPDH (mAb, Proteintech, catalog no. 60004-1-Ig, 1:10000); β-Actin (mAb, Proteintech, catalog no. 66009-1-Ig, 1:10000); HRP-conjugated Affinipure Goat Anti-Mouse IgG(H+L) (Proteintech, catalog no. SA00001-1, 1:10000); HRP-conjugated Affinipure Goat Anti-Rabbit IgG(H+L) (Proteintech, catalog no. SA00001-2, , 1:10000); Goat Anti-Rat IgG H&L (Alexa Fluor® 647) preadsorbed (Abcam, catalog no. ab150167, 1:250); Goat Anti-Rabbit IgG H&L (Alexa Fluor® 568) (Abcam, catalog no. ab175471, 1:250); Goat Anti-Mouse IgG H&L (Alexa Fluor® 488) (Abcam, catalog no. a b150113, 1:250).

Validation

All antibodies were well-recognized clones in the field and validated by the manufacturers. These antibodies are further validated and routinely used in our lab.

ADAM10 (pAb, Abcam, catalog no. ab1997) has been cited at least 120 times and validated for use in WB application (https://www.abcam.cn/products/primary-antibodies/adam10-antibody-ab1997.html).

APP, C-Terminal (pAb, Sigma, catalog no. A8717) has been cited at least 363 times and validated for use in WB application (https://www.sigmaaldrich.com/US/en/product/sigma/a8717?srsltid=AfmBOorbfrx9bxZ3DfYihi7_pw2lLV4iKmkFgR1GCQF3j6cfmlh57eXp).

β-Amyloid, 1-16 [6E10] (mAb, BioLegend, catalog no. 803014) has been cited at least 84 times and validated for use in WB and IHC-F applications (https://www.biolegend.com/en-ie/products/anti-beta-amyloid-1-16-antibody-10998).

BACE1 (pAb, Abcam, catalog no. ab2077) has been cited at least 91 times and validated for use in WB application (https://www.abcam.cn/products/primary-antibodies/bace1-antibody-ab2077.html).

BACE1 [EPR3956] (mAb, Abcam, catalog no. ab108394) has been cited at least 67 times and validated for use in WB application (https://www.abcam.cn/products/primary-antibodies/bace1-antibody-epr3956-ab108394.html).

BACE1 [EPR19523] (mAb, Abcam, catalog no. ab183612) has been cited at least 34 times and validated for use in WB and IHC applications (https://www.abcam.cn/products/primary-antibodies/bace1-antibody-epr19523-ab183612.html).

PCBP2 [EPR14858] (mAb, Abcam, catalog no. ab184962) has been cited at least 7 times and validated for use in WB and ICC/IF applications (https://www.abcam.cn/products/primary-antibodies/pcbp2hnrnp-e2-antibody-epr14858-ab184962.html).

RENT1/hUPF1 [EPR4681] (mAb, Abcam, catalog no. ab109363) has been cited at least 25 times and validated for use in WB and ICC/IF applications (https://www.abcam.cn/products/primary-antibodies/rent1hupf1-antibody-epr4681-ab109363.html).

Phospho-Upf1 (Ser1127) (pAb, Sigma, catalog no. 07-1016) has been cited at least 27 times and validated for use in WB application (https://www.sigmaaldrich.com/US/en/product/mm/071016?srsltid=AfmBOoqxUDVn2NjAjIrjfMTRtU8BPHLD4tWAjKd7u_-mSypOoFdVjCmc).

TIA1 (mAb, Santa Cruz, catalog no. sc-166247) has been cited at least 38 times and validated for use in IF application (https://www.scbt.com/p/tia-1-antibody-g-3?srsltid=AfmBOoqKB3oFxUdZu-gV2MkjkpaNfBUvbHNlPzTrQuI3NYUnuOXiJBDt).

DDX6 (mAb, Santa Cruz, catalog no. sc-376433) has been cited at least 15 times and validated for use in IF application (https://www.scbt.com/p/rck-antibody-e-12).

DCP1a (mAb, Santa Cruz, catalog no. sc-100706) has been cited at least 38 times and validated for use in IF application (https://www.scbt.com/p/dcp1a-antibody-56-y).

TOMM20 [EPR15581-39] (mAb, Abcam, catalog no. ab283317) has been cited at least 16 times and validated for use in IF application (https://www.abcam.cn/products/primary-antibodies/tomm20-antibody-epr15581-39-mouse-igg1-chimeric-ab283317.html).

NeuN [1B7] (mAb, Abcam, catalog no. ab104224) has been cited at least 877 times and validated for use in ICC/IF application (https://www.abcam.cn/products/primary-antibodies/neun-antibody-1b7-neuronal-marker-ab104224.html).

Iba1 [EPR16589] (mAb, Abcam, catalog no. ab283346) has been cited at least 18 times and validated for use in ICC/IF application (https://www.abcam.cn/products/primary-antibodies/iba1-antibody-epr16589-rat-igg2a-chimeric-ab283346.html).
GFAP [EPR1034Y] (mAb, Abcam, catalog no. ab279291) has been cited at least 7 times and validated for use in ICC/IF application (https://www.abcam.cn/products/primary-antibodies/gfap-antibody-epr1034y-rat-igg2a-chimeric-ab279291.html).
INTS1(pAb, Proteintech, catalog no. 31428-1-AP) has been validated through WB analysis in lysates from various cell types (https://www.ptgcn.com/products/INTS1-Antibody-31428-1-AP.htm). This paper presents validation results obtained through knockdown experiments (Fig. 7b).
Histone Macro-H2A.1 (mAb, Invitrogen, catalog no. MA5-24696) has been verified by relative expression to ensure that the antibody binds to the antigen stated (https://www.thermofisher.cn/antibody/product/MA5-24696.html?CID=AFLCA-MA5-24696).This paper presents validation results obtained through knockdown experiments (Fig. 7c).
TUBGCP2 (pAb, Proteintech, catalog no. 25856-1-AP, 1:1000) has been cited at least 1 times and validated for use in WB and ICC/IF applications (https://www.ptgcn.com/products/TUBGCP2-Antibody-25856-1-AP.htm). This paper presents validation results obtained through knockdown experiments (SFig. 10c).
HSP40 (mAb, Santa Cruz, catalog no. sc-398766, 1:50) has been cited at least 12 times and validated for use in WB and ICC/IF applications (https://www.scbt.com/zh/p/hsp-40-antibody-b-3).
DNAJC16 (pAb, FineTest, catalog no. FNab02461, 1:1000) (https://www.amsbio.com/anti-dnajc16-antibody-amsfnab02461). This paper presents validation results obtained through knockdown experiments (SFig. 10d).
Flag [DYKDDDDK tag] (mAb, Proteintech, catalog no. 66008-4-Ig) has been cited at least 273 times and validated for use in WB, IP, CoIP and RIP applications (https://www.ptgcn.com/products/Flag-tag-Antibody-66008-4-Ig.htm#product-information).
GFP (mAb, Proteintech, catalog no. 66002-1-Ig) has been cited at least 477 times and validated for use in WB, IP and CoIP applications (https://www.ptgcn.com/products/eGFP-Antibody-66002-1-Ig.htm).
mCherry (mAb, Proteintech, catalog no. 68088-1-Ig, 1:2000) has been verified by relative expression to ensure that the antibody binds to the antigen stated (https://www.ptglab.com/products/mCherry-Antibody-68088-1-Ig.htm).
GAPDH (mAb, Proteintech, catalog no. 60004-1-Ig) has been cited at least 11243 times and validated for use in WB application (https://www.ptgcn.com/products/GAPDH-Antibody-60004-1-Ig.htm).
β-Actin (mAb, Proteintech, catalog no. 66009-1-Ig) has been cited at least 6262 times and validated for use in WB application (https://www.ptgcn.com/products/Pan-Actin-Antibody-66009-1-Ig.htm).
HRP-conjugated Affinipure Goat Anti-Mouse IgG(H+L) (Proteintech, catalog no. SA00001-1) has been cited at least 6866 times and validated for use in WB application (https://www.ptgcn.com/products/HRP-conjugated-Affinipure-Goat-Anti-Mouse-IgG-H-L-secondary-antibody.htm).
HRP-conjugated Affinipure Goat Anti-Rabbit IgG(H+L) (Proteintech, catalog no. SA00001-2) has been cited at least 9296 times and validated for use in WB application (https://www.ptgcn.com/products/HRP-conjugated-Affinipure-Goat-Anti-Rabbit-IgG-H-L-secondary-antibody.htm).
Goat Anti-Rat IgG H&L (Alexa Fluor® 647) preadsorbed (Abcam, catalog no. ab150167) has been cited at least 55 times and validated for use in IF application (https://www.abcam.cn/products/secondary-antibodies/goat-rat-igg-hl-alexa-fluor-647-preadsorbed-ab150167.html).
Goat Anti-Rabbit IgG H&L (Alexa Fluor® 568) (Abcam, catalog no. ab175471) has been cited at least 197 times and validated for use in IF application (https://www.abcam.cn/products/secondary-antibodies/goat-rabbit-igg-hl-alexa-fluor-568-ab175471.html).
Goat Anti-Mouse IgG H&L (Alexa Fluor® 488) (Abcam, catalog no. ab150113) has been cited at least 1264 times and validated for use in IF application (https://www.abcam.cn/products/secondary-antibodies/goat-mouse-igg-hl-alexa-fluor-488-ab150113.html).

Eukaryotic cell lines

Policy information about cell lines and Sex and Gender in Research

Cell line source(s)	SH-SY5Y cells were purchased from the National Collection of Authenticated Cell Cultures, catalog no. SCSP-5014. HEK293T cells were purchased from ATCC, catalog no. CRL-3216. The HEK293T-APP and SH-SY5Y-APP cell lines were reported in previous studies(Chen, Luo et al. 2021, Zhou, Tang et al. 2023).
Authentication	Authentication and validation information can be found on the sources' website. The SH-SY5Y cell line most frequently used in this paper was validated by STR profiling, showing an exact match with the SH-SY5Y line in the ExPASy STR database based on 13 core STR loci. Other cell lines used in this study were not authenticated.
Mycoplasma contamination	All cell lines in our laboratory are routinely tested for mycoplasma contamination and cells used in this study are negative for mycoplasma.
Commonly misidentified lines (See ICLAC register)	No cell line used in the paper is listed in ICLAC database.

Animals and other research organisms

Policy information about studies involving animals; ARRIVE guidelines recommended for reporting animal research, and Sex and Gender in Research

Laboratory animals	APP/PS1 mice (APPswe, PSEN1dE9, B6C3, #034829-JAX) and 5×FAD mice (APPSwFlLon, PSEN1*M146L*L286V, B6SJL, #034840-JAX) transgenic mice were purchased from Jackson Laboratory or GENEANDPEACE, respectively. Mice were maintained in a specific-pathogen -free facility in individually ventilated cages (3-5 per cage) on a 12-h light/12-h dark cycle, at an ambient temperature of 22 ± 2℃ and relative humidity of 40-60%. Standard chow and water were provided ad libitum, with nesting materials and environmental enrichment. Animals were acclimated for at least 7 days before experimentation. Wild-type (WT) mice of the same genetic background were identified by genotyping; only adult male mice were included and randomly assigned to experimental groups. Mice with exact n and ages reported in the figure legends were used in this study.

Wild animals	The study did not involve wild animals.
Reporting on sex	In this study, we only utilized male mice to circumvent potential confounding effects arising from cyclical hormonal fluctuations associated with the female estrous cycle.
Field-collected samples	The study did not involve samples collected from field.
Ethics oversight	All animal experimental protocols were approved by Institutional Animal Care and Use of Chongqing Medical University (approval no. IACUCCQMU-2024-0023) in accordance with international standards.

Note that full information on the approval of the study protocol must also be provided in the manuscript.

Plants

Seed stocks	*Report on the source of all seed stocks or other plant material used. If applicable, state the seed stock centre and catalogue number. If plant specimens were collected from the field, describe the collection location, date and sampling procedures.*
Novel plant genotypes	*Describe the methods by which all novel plant genotypes were produced. This includes those generated by transgenic approaches, gene editing, chemical/radiation-based mutagenesis and hybridization. For transgenic lines, describe the transformation method, the number of independent lines analyzed and the generation upon which experiments were performed. For gene-edited lines, describe the editor used, the endogenous sequence targeted for editing, the targeting guide RNA sequence (if applicable) and how the editor was applied.*
Authentication	*Describe any authentication procedures for each seed stock used or novel genotype generated. Describe any experiments used to assess the effect of a mutation and, where applicable, how potential secondary effects (e.g. second site T-DNA insertions, mosiacism, off-target gene editing) were examined.*

Flow Cytometry

Plots

Confirm that:

- [x] The axis labels state the marker and fluorochrome used (e.g. CD4-FITC).
- [x] The axis scales are clearly visible. Include numbers along axes only for bottom left plot of group (a 'group' is an analysis of identical markers).
- [x] All plots are contour plots with outliers or pseudocolor plots.
- [x] A numerical value for number of cells or percentage (with statistics) is provided.

Methodology

Sample preparation	For each sorting experiment, cells were cultured to approximately 80-90% confluence in three 15-cm dishes. Cells were pelleted in PBS by centrifugation at 1,000 × g for 3 min at RT and immediately flash-frozen in liquid nitrogen overnight. Unless otherwise stated, the following steps were conducted at 4°C or on ice. Cell pellets were resuspended in lysis buffer (50 mM Tris [pH 7.4], 1 mM EDTA, 150 mM NaCl, 0.2% Triton X-100), containing 65 U/mL RNase inhibitor and EDTA-free protease inhibitor cocktail. To facilitate lysis, the extracts were passed 20 times through a 25G syringe needle, with a total incubation time of 20 min. Lysates were spun at 200 x g for 5 min to remove nuclei. To digest remaining DNA contaminants, the supernatants were treated with RQ1 RNase-free DNase for 30 min at RT, and then centrifuged at 10,000 x g for 7 min. The resulting pellets were resuspended in 2 mL of lysis buffer, and the organelle-enriched fraction obtained at 10,000 x g was designated as the pre-sorted fraction. From this fraction, PCBP2-biomolecular condensates were sorted on a cell sorter (Bigfoot, Invitrogen) equipped with a 100 µm nozzle and operating at a pressure of 30 psi. Particles were identified based on their forward-scattered light (FSC) and mCherry fluorescence, utilizing a 561 nm excitation laser and a 615/24 nm bandpass filter. The sorting window for PCBP2-biomolecular condensates was specifically set to exclude mCherry-SH-SY5Y fluorescent particles while retaining mCherry-labeled PCBP2 condensates. The mCherry-PCBP2 fraction represented 8-10% of total events and was subsequently collected.
Instrument	Invitrogen™ Bigfoot Spectral Cell Sorter
Software	Flow cytometry was collected and analyzed with SQ Software (Invitrogen) and FlowJo version 10.8.1, respectively.
Cell population abundance	When cells were sorted or enriched, the purity was confirmed by flow cytometry and in each case the purity was above 90% .
Gating strategy	Particles were selected using FSC/SSC gates such that 95% of all events were within the range. mCherry (Fig. 2b) serves as a control for non-PCBP2 particles. Sorting window for mCherry-PCBP2 particlesis delineated by black squares (Fig. 2c).

- [x] Tick this box to confirm that a figure exemplifying the gating strategy is provided in the Supplementary Information.
